# Genetic Diversity of a Heat Activated Channel—TRPV1 in Two Desert Gerbil Species with Different Heat Sensitivity

**DOI:** 10.3390/ijms24119123

**Published:** 2023-05-23

**Authors:** Bing Wang, Xue-Ying Zhang, Shuai Yuan, He-Ping Fu, Chen-Zhu Wang, De-Hua Wang

**Affiliations:** 1 State key Laboratory of Integrated Management of Pests Insects and Rodents, Institute of Zoology, Chinese Academy of Sciences, Beijing 100101, China; 2 CAS Center for Excellence in Biotic Interactions, University of Chinese Academy of Sciences, Beijing 100049, China; 3 College of Grassland, Resources and Environment, Inner Mongolia Agricultural University, Hohhot 010018, China; 4 School of Life Sciences, Shandong University, Qingdao 266237, China

**Keywords:** heat sensitivity, transient receptor potential vanniloid 1 (TRPV1), genetic diversity, Mongolian gerbils (*Meriones unguiculatus*), mid-day gerbils (*Meriones meridianus*)

## Abstract

Heat sensation and tolerance are crucial for determining species’ survival and distribution range of small mammals. As a member of the transmembrane proteins, transient receptor potential vanniloid 1 (TRPV1) is involved in the sensation and thermoregulation of heat stimuli; however, the associations between animal’s heat sensitivity and TRPV1 in wild rodents are less studied. Here, we found that Mongolian gerbils (*Meriones unguiculatus*), a rodent species living in Mongolia grassland, showed an attenuated sensitivity to heat compared with sympatrically distributed mid-day gerbils (*M. meridianus*) based on a temperature preference test. To explain this phenotypical difference, we measured the TRPV1 mRNA expression of two gerbil species in the hypothalamus, brown adipose tissue, and liver, and no statistical difference was detected between two species. However, according to the bioinformatics analysis of TRPV1 gene, we identified two single amino acid mutations on two TRPV1 orthologs in these two species. Further Swiss-model analyses of two TRPV1 protein sequences indicated the disparate conformations at amino acid mutation sites. Additionally, we confirmed the haplotype diversity of TRPV1 in both species by expressing TRPV1 genes ectopicly in *Escherichia coli* system. Taken together, our findings supplemented genetic cues to the association between the discrepancy of heat sensitivity and the functional differentiation of TRPV1 using two wild congener gerbils, promoting the comprehension of the evolutionary mechanisms of the TRPV1 gene for heat sensitivity in small mammals.

## 1. Introduction

For animals, species’ survival and distribution are largely restricted by ecological factors, such as photoperiod, water, food availability and temperature [1,2]. With the procession of global warming, organisms are under a more impending selective pressure from heat environment due to their constraints by physiological features [3]. When exposed to heat pressure, mammals need to keep their core body temperature stable, to provide a suitable condition for biochemical reactions within their bodies [4,5]. The external signals of high temperature are transmitted to the hypothalamic thermoregulatory center through warm-sensitive neurons, which commands the thermoeffectors to increase heat loss and decrease heat production. Thus, the heat sensitivity during this physiological process plays a decisive role in the development of mammals [6,7,8,9,10]. However, we still know little about the sensitivity of the thermosensory system to heat.

Transient receptor potential (TRP) channels are heavily involved in the sensation of various thermal and chemical stimuli in mammals and participate in numerous physiological processes [11,12,13]. TRPs are divided into seven subfamilies according to the different activation conditions and structural homology: TRPC (canonical), TRPA (ankyrin), TRPV (vanilloid), TRPM (melastatin), TRPML (mucolipin), TRPP (polycystin), and TRPN (non-mechanosensitive channels in insects) [14,15,16]. TRPV1 is a member of TRPV subfamily, and is the first characterized molecular target responsible for thermal regulation [17]. As a transmembrane protein, TRPV1 can be modulated by both exogenous and endogenous regulators, such as vanilloids, flavonoids, 20-hydroxyeicosatetraenoic acid (20-HETE), endocannabinoids N-acylamides (N-acyl GABA), and glycerophospholipids (lysophosphatidic acid, LPA), which are of great importance to animals’ physiological function [18,19,20,21]. Researches indicated that the deletion of *trpv1* in mice did not abolish pain sensitivity in general but diminished sensitivity to noxious temperatures more than 50 °C [22,23,24]. The maximum heat activation of TRPV1 in different vertebrates, such as the thirteen-lined ground squirrel (*Ictidomys tridecemlineatus*) and wild Bactrian camel (*Camelus ferus*), with distinct amino acid sequences in the pore domain to be tuned to match their habitat temperature [25]. TRPV1 is not only essential for thermosensation, but also plays an important role in thermoregulation. The activation of TRPV1 by capsaicin clearly strengthened both heat production and dissipation in mice [26]. With the development of cryogenic electron microscopy, we also know much about the functionally critical regions in TRPV1 [27,28,29], but there are still gaps in knowledge regarding the role of TRPV1 in heat sensitivity.

Mongolian gerbils (*Meriones unguiculatus*) and mid-day gerbils (*M. meridianus*) are closely related species and are sympatrically distributed in Inner Mongolia, where there is a large difference in ambient temperature as the maximum annual temperature difference reached 86 °C. In their distributions, *M. meridianus* showed critically nocturnal activity, while *M. unguiculatus* preferred to be diurnal [30,31]. These different activity patterns contributed to their sympatry but also required *M. unguiculatus* to have more tolerance to heat. Previous data showed a significant difference in the thermal neutral zone (TNZ) between these species. The TNZ of *M. unguiculatus* was 26.5–38.9 °C, while *M. meridianus* was 25.5–30.1 °C, which suggests that *M. unguiculatus* possessed more tolerance to heat [10,31]. Many tissues participated in the thermoregulatory homeostasis for small mammals exposed to heat stress, including brown adipose tissue (BAT), liver and brain, and *M. unguiculatus* showed less alteration when faced with heat than *M. meridianus* at these levels [8,9,10,32]. Interestingly, administration of capsaicin, an agonist of TRPV1, significantly lowered surface body temperature, and changed the preference to different temperatures in *M. unguiculatus* [33,34]; however, the genetic basis for difference in heat adaptation in these two congeners still remains poorly understood.

To test the hypothesis that the genetic diversity of *trpv1* may be associated with the difference in heat sensitivity between species, we firstly measured the basal physiological traits of *M. unguiculatus* and *M. meridianus* captured in the wild. Then, the TRPV1 mRNA expression pattern was analyzed with quantitative, real-time PCR (qPCR), and the next-generation sequencing of *M. meridianus* genome was performed to supply more accurate genetic information for TRPV1. Lastly, we conducted PCR sequencing and ectopic expression of the TRPV1 gene in competent *Escherichia coli* to further identify the TRPV1 genetic diversity in these species. This study improved our understanding on the heat sensitivity difference between species. 

## 2. Results

### 2.1. Morphological and Physiological Traits of M. unguiculatus and M. meridianus 

**Figure 1:**
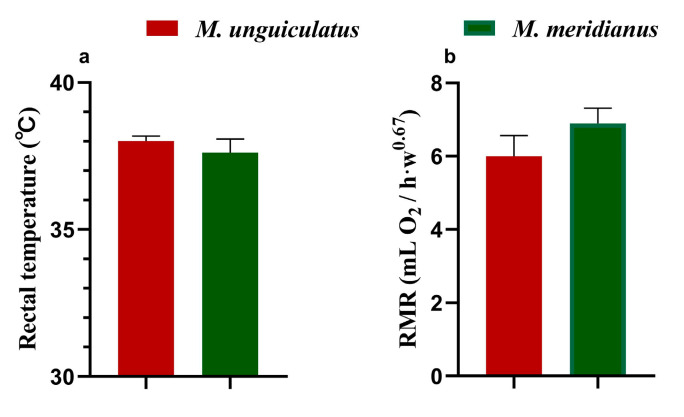
Physiological traits of *M. unguiculatus* and *M. meridianus*. (**a**) Rectal temperature. (**b**) Resting metabolic rate (RMR).

To monitor whether the animals used in this study were at the same state of growth, the morphological traits of animals were recorded. There was no significant difference in body length or tail length between *M. unguiculatus* and *M. m eridianus*. The body mass, ear length and metapodial length were higher in *M. meridianus* than in *M. unguiculatus* (Table 1). For physiological traits, the rectal temperature (38.0 ± 0.2 °C vs. 37.6 ± 0.5 °C) and resting metabolic rate (RMR, 5.996 ± 0.567 mLO_2_/h·w^0.67^ vs. 6.897 ± 0.411 mLO_2_/h·w^0.67^) between *M. unguiculatus* and *M. meridianus* showed no difference (Figure 1).

### 2.2. Temperature Preference Trials

**Figure 2:**
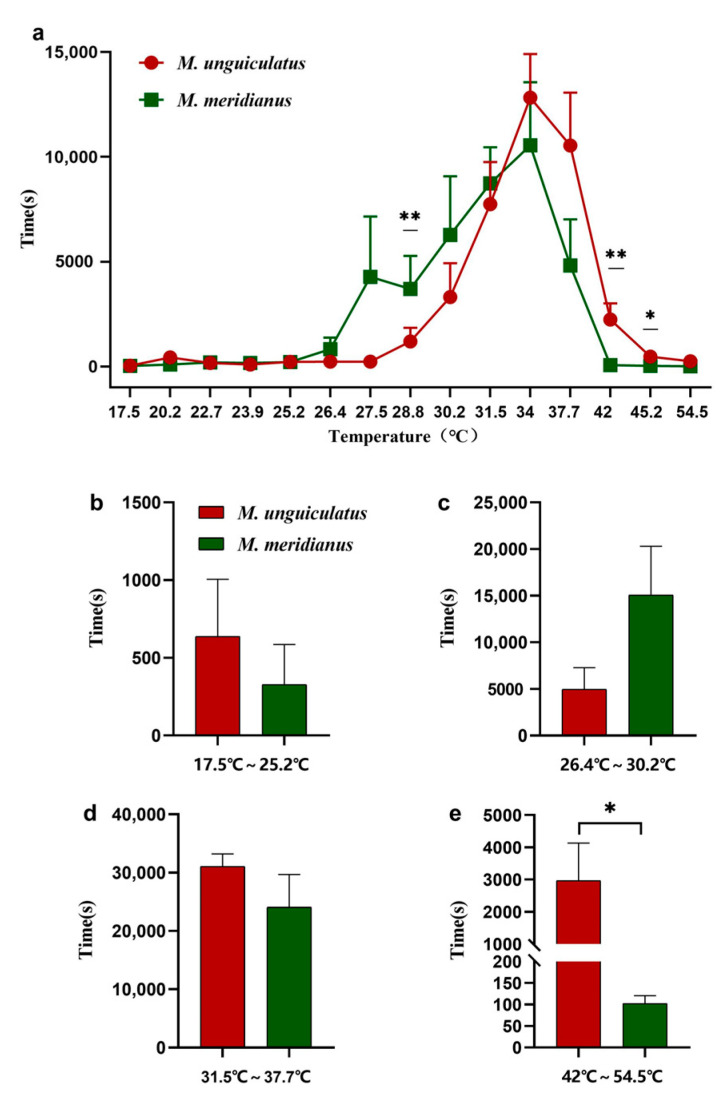
The duration that two gerbil species spent at different temperature areas. (**a**) Duration at all recording sites. Duration (**b**) below thermal neutral zone (TNZ), (**c**) in the lower part of TNZ, (**d**) in the upper part of TNZ and (**e**) over TNZ. * *p* < 0.05; ** *p* < 0.01.

Heat sensitivity and tolerance of two congeners were tested by temperature preference test. The temperature gradient area was divided into four parts in consideration of the gerbils’ TNZ: below TNZ (17.5–25.2 °C), the lower part of TNZ (26.4–30.2 °C), the upper part of TNZ (31.5–37.7 °C), and over TNZ (42–54.5 °C). The time animals spent at each area was significantly different as the time spent within TNZ exceeded the time spent beyond TNZ (F_14,189_ = 14.84, *p* < 0.01, Figure 2a). Additionally, the two species spent almost the same time at the upper part within TNZ, but *M. unguiculatus* spent 67% less time than *M. meridianus* at the lower part within TNZ (Figure 2b–d). Remarkably, the duration *M. unguiculatus* spent over TNZ exceeded that of *M. meridianus* significantly (t = 2.286, df = 13, *p* = 0.04, Figure 2e). These data clearly showed that *M. unguiculatus* was equipped with a lower sensitivity and better tolerance to higher temperature than *M. meridianus*. 

### 2.3. The mRNA Expression Pattern of TRPV1 Gene in Two Species

**Figure 3:**
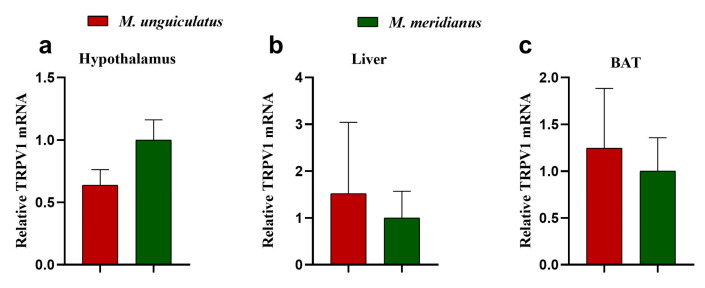
The relative TRPV1 mRNA expression in the hypothalamus (**a**), liver (**b**), and brown adipose tissue (BAT) (**c**) in two gerbil species.

To test whether there is a species difference in TRPV1 expression in the organs related with thermoregulation, we detected the TRPV1 mRNA expression from the hypothalamus (t = 1.746, df = 12, *p* = 0.106), liver (t = 0.336, df = 12, *p* = 0.743), and BAT (t = 0.321, df = 11, *p* = 0.755) through qPCR. There was no statistical significance between species in these three organs (Figure 3).

### 2.4. Homology Analysis of utrpv1 (trpv1 of M. unguiculatus) and mtrpv1 (trpv1 of M. meridianus)

**Figure 4:**
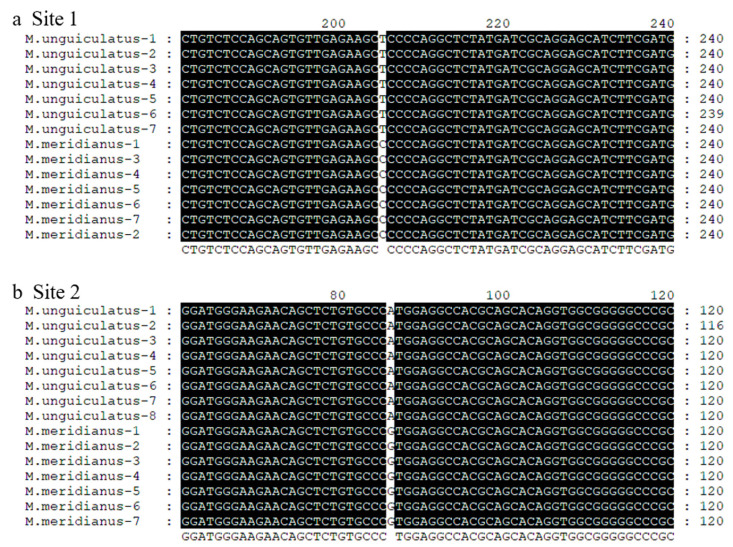
The gene sequence near two missense mutation sites validated in two gerbil species. Site 1 from T in *u*TRPV1 (*M. unguiculatus*) to C in *m*TRPV1 (*M. meridianus*) (**a**) and site 2 from A in *u*TRPV1 to G in *m*TRPV1 (**b**).

For the identification of genetic characteristics of *trpv1*, we launched the sequencing and analysis of two gerbils. The full cDNA sequence of *utrpv1* and *mtrpv1* were both 2523 bp (including three base pairs for termination codon), and 99.28% were identical with each other (18 mutation sites) (Table 2). Among these mutation sites, only two were missense, causing alterations in protein encoding (V610-M610, P109-L109) (Table 3). Certainly, these two missense mutation sites were then validated by PCR amplification (Figure 4). The full coding DNA sequence of *M. meridianus* was shown in Table S1.

### 2.5. Structural Prediction of uTRPV1 and mTRPV1

**Figure 5:**
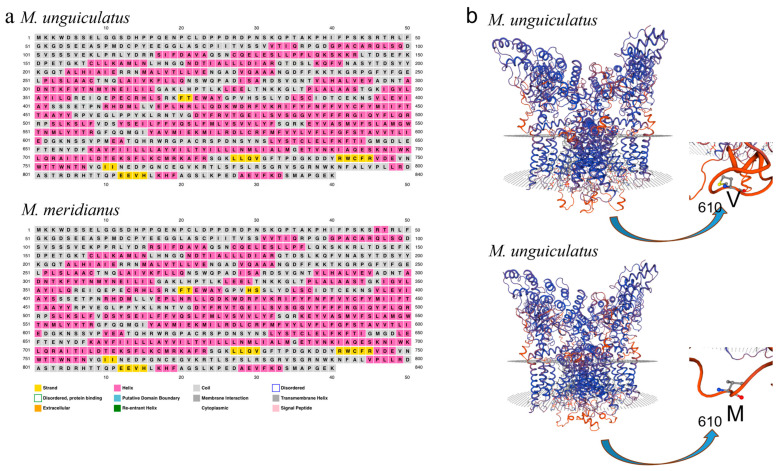
The amino acid substitution sites and predicted structure of TRPV1 between two gerbil species. The secondary structure prediction of TRPV1 proteins from two species (**a**), and partial topology conformations of TRPV1 at V610 and M610 (**b**).

According to the structural prediction, *u*TRPV1 and *m*TRPV1 showed particularly high conservation (99.76%) with each other (Table 2). Even so, the amino acid folding preference prediction suggests that the first amino acid mutation prefers to be located in the transmembrane helix (Figure 5a), which is an important domain for the function of TRPV1. Additionally, the second substitutions of V-M may alter the ultrastructure of TRPV1 in its intracellular domain (Figure 5b). 

### 2.6. Cloning of TRPV1 and Ectopic Expression in E. coli System

**Figure 6:**
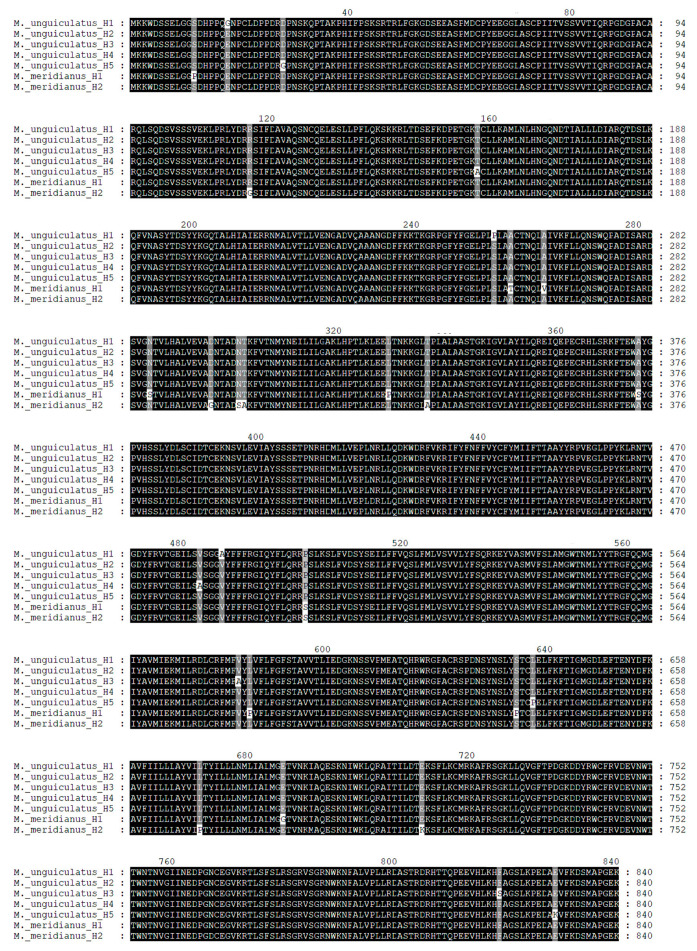
Diversity of haplotypes in two gerbil species. Five haplotypes for *M. unguiculatus* and two for *M. meridianus* were displayed. Single nucleotide polymorphisms were marked with different background color.

To verify the biological characteristic of two TRPV1 orthologs in vivo, we obtained cDNAs of TRPV1 from *M. unguiculatus* and *M. meridianus* by PCR and subsequently transfected them into *E. coli* system. After reproduction, the bacterial suspension of transfected *E. coli* was sequenced. Data showed haplotype diversity in both two congener species and no distinct significance was found on this diversity (Figure 6). 

## 3. Discussion

Due to the ability of high reproducibility and adaptation to changing environments, rodents were generally considered as the important indicator species for the health of local desert ecosystem [35]. However, with global warming and excess land reclamation, wild habitats for animals have been gradually destroyed, which has altered the existing distribution pattern [36,37,38]. *M. unguiculatus* and *M. meridianus* were the typical rodents, which took an important part in the comparable fragile ecosystem of desert [38,39]. The comparative study in thermal biology of these two gerbils was of great necessity for the forecast of the impacts of global warming on population dynamics. Similarly, the rectal temperature and RMR suggested their convergent strategy of energy expenditure, but *M. unguiculatus* was more tolerant to heat than *M. meridianus,* as indicated by temperature preference test, which gives the former an advantage in facing global warming [40]. This is undoubtedly conducive for the survival of *M. unguiculatus*. To clarify the internal determinants on animals’ thermal sensitivity and tolerance, increasingly more data are highlighting the importance of thermos-TRPs, of which the activation threshold is closely related with its sensitivity to temperature.

TRP channels are parts of the transmembrane proteins located on cell membranes, and are responsible for the signal conversion from external stimuli into cell membrane potential change, which may alter the concentration of the most important secondary messenger—Ca^2+^ [11]. TRPV1 was named because of the activation by vanilloid-like compounds. In addition to this, TRPV1 can also be activated by numerous factors, including temperature. Consensus had been achieved that the sensitivity of TRPV1 orthologs to heat could be mediated by a single amino acid [41]. As a transmembrane protein for signal transduction, TRPV1 sensitivity can be altered by minimal change in conformation and structure. The high sensitivity to oxidants of human TRPV1 was probably explained by the special conformation of the finger 3 loop in the N-terminal ankyrin repeat domain [42]. When the pore turret of TRPV1 was mutated with eight residues left, the sensitivity of TRPV1 to heat was declined 10-fold [43]. Other TRP channels were also confirmed to have this functional plasticity of transmembrane protein between species. For example, TRPM8, which is in charge of cold activation, was tuned to suppress cold sensitivity with a mutation in amino acid sequences in penguins [44]. In our study, two missense mutations were identified to provide a potential explanation on the imparity of heat sensitivity between two congeners. BAT and liver are two important metabolic organs, and the hypothalamus is the center for controlling the activity of these two organs. Thus, these three organs are closely related with the heat sensitivity in small mammals; however, the mRNA expression pattern of TRPV1 indicates that the characteristic of heat sensitivity in animals is not related to the quantity of TRPV1 protein, for *M. unguiculatus* and *M. meridianus* at least. However, further genetic analysis of TRPV1 showed that these two mutation sites may result in distinct protein conformations according to model predicting. The extracellular domain of TRPV1 may participate in the regulation of temperature sensitivity where the first substitution occurred. Additionally, the prediction of the second mutation sites also showed different physicochemical properties. 

Furthermore, haplotype diversity is also an important component of genetic trait for mammals [45,46]. According to the sequence analysis of transducted *E. coli*, higher levels of gene polymorphism at *trpv1* was found in *M. unguiculatus* than in *M. meridianus*, at least in our present data. It had been indicated that under the xeric environments, the *M. meridianus* population was more dependent on annual mean temperature compared to sympatrically distributed *M. unguiculatus* as well as other species, such as Northern three-toed jerboa (*Dipus sagitta*)*,* Andrew’s three-toed jerboa (*Stylodipus andrewsi*)*,* Siberian jerboa (*Qrientallactaga sibirica*)*,* desert hamster (*Phodopus roborovskii*)*,* striped dwarf hamster (*Cricetulus barabensis*)*,* Eversman’s hamster (*Allocricetulus eversmanni*)*,* and Alashan ground squirrel (*Spermophilus alaschanicus*) [39]. High genetic diversity is one of the most powerful indicators of a population’s evolutionary potential. Therefore, *M. meridianus* may be suffering more than *M. unguiculatus* under the selection pressure from changing environments [46,47,48].

The study of the associations between genetic diversity in TRPV1 and different heat sensitivities in wild animals is of vital significance in understanding the strategy that animals follow to deal with high temperature, especially under the increasing threat of global warming. Indeed, animals’ adaptation to heat environment is a relatively complex physiological process, including the balance between heat production and heat dissipation, and between energy intake and energy expenditure [49,50,51]. Heat sensitivity was the first step and featured in this process, but the basic influencing factor on the heat tolerance diversity in animals were far from a conclusion. In our study, we identified two fixed mutation sites on the heat-activated TRPV1 channel, which resulted in the substitution of amino acid between a pair of wild animal models with the closest relationship but different heat sensitivity. We also confirmed the haplotype polymorphism within taxa in both species of the TRPV1 gene. However, as TRPV1 can be stimulated by plenty of biological signals, more details are needed to verify the associations between genetic diversity of TRPV1 and the difference in heat sensitivity in animals. 

## 4. Materials and Methods

### 4.1. Animals

The adult *M. unguiculatus* (4 males and 7 females) and *M. meridianus* (4 males and 4 females) were all live-captured from the Gobi Desert of Inner Mongolia in August 2021. They were placed in an indoor environment with natural photoperiod and fed with a standard rat pellet chow (Beijing KeAo Bioscience Co., Beijing, China) and peanuts, with water provided *ad libitum*. All the animals were housed individually in plastic net cages (40 cm × 40 cm × 60 cm) with shading box filled with sand for bedding during our experiment. 

All animal procedures were approved by the Animal Care and Use Committee of the Institute of Zoology, Chinese Academy of Sciences (IOZ-IACUC-2021-194).

### 4.2. Measurement of Rectal Temperature and Metabolic Rate

The rectal temperature was measured with a digital thermometer (TES-1310, TES, Taiwan, China). The probe of the thermometer was lubricated with glycerin, and then inserted 3 cm into the rectum and the highest stable value was taken within 30 s.

RMR was calculated as oxygen consumption rate using a multiple-channel FMS open flow system (Sable System, Las Vegas, NV, USA) with each gerbil placed into a pellucid plastic chamber (2.7 L). The flow rate of fresh air pumped into system was 500–600 mL/min, and the air was dried by a non-chemical gas drier before entering the chamber. The air leaving the chamber was dried again and was transported to the oxygen analyzer with a flow rate as 100 mL/min. The change of air composition was transducted into digital signals by a computer connected with an analogue-to-digital converter. The three consecutive lowest oxygen consumption volume within 30 min was picked during a 3 h total measurement, and the average volume was recognized as the RMR [52,53].

### 4.3. Temperature Preference Test

The apparatus consists of one plastic cage (35 cm × 25 cm × 20 cm) filled with an ice-water mixture, stabilizing at 1.8 °C, and a thermostatic water bath set to 85 °C. These two cages were placed next to each other, separated by a board of polyethylene foam, and covered with an opaque steel plate. The temperature gradient plate was formed and the temperatures were measured by thermometers. The apparatus was boarded up with wood planks, where there were 15 infrared thermometers distributed uniformly at the bottom to record the time gerbils stay at each area. The room temperature was maintained at 23 ± 1 °C. Subjects were placed in the middle line of the plastic plate initially and allowed to roam freely. Before time recording, subjects were given 30 min to get accustomed to this apparatus, and then the thermometers were plugged in. Subject’s time spent in each area of the plate was recorded for totally 150 min. The duration of time spent in the heated area (near to the water bath) was used as an index of hot tolerance [34].

After the measurement of temperature preference, the animals were anesthetized with isoflurane firstly, to relieve the animal’s suffering and then sacrificed. Body mass, body length, ear length, metapodial length, and tail length were measured. The liver, hypothalamus, and BAT were excised quickly and frozen in liquid nitrogen immediately. All the tissues were stored at −80 °C.

### 4.4. qPCR Analysis of TRPV1

TRIzol reagent (R401-01, Vazyme, Nanjing, China) was used for total RNA isolation according to the protocol provided by the manufacturer. Then, total RNA was purified and reverse-transcribed to cDNA using cDNA Synthesis (+gDNA wiper) Kit (R312-01, Vazyme). Quantitative real-time PCR was carried out using Taq Pro Universal SYBR qPCR Master Mix (Q712-02, Vazym, Nanjing, China) on MX3000P (Stratagene, CA, USA). Glyceraldehyde-3-phosphate dehydrogenase (GAPDH) served as the reference gene [8]. Three biological replications were performed for each sample. The 2^−ΔΔCt^ method was used to calculate the relative expression of the target gene. Primers (Table S2) used in qPCR were all designed by primer-blast on NCBI (https://www.ncbi.nlm.nih.gov/tools/primer-blast/index.cgi?LINK_LOC=BlastHome (accessed on 13 May 2022)), referring to the *M. unguiculatus* genome sequence on NCBI (https://www.ncbi.nlm.nih.gov/nuccore/ (accessed on 13 May 2022)).

### 4.5. Next-Generation Sequencing and TRPV1 Gene Analysis of M. meridianus

The genome of *M. unguiculatus* has been published on NCBI, so we simply launched the next-generation sequencing for *M. meridianus*. The animal used for next-generation sequencing was an adult offspring of wild-captured *M. meridianus* from the same Gobi Desert of Inner Mongolia with those used for other measurements. The blood was collected from the infraorbital vein via capillary tube, which is a non-fatal method of blood collection. The quality of blood sample was detected, and then a DNA library was established and sequenced with Illumina NovaSeq 6000 platform at Novogene Co., Ltd., Beijing, China. Raw data were obtained and further processed to get clean data with removal of low-quality paired reads. Then, a SNP summary file between *M. unguiculatus* and *M. meridianus* was generated using BWA (https://github.com/lh3/bwa (accessed on 27 June 2022)), Samtools (https://github.com/samtools/ (accessed on 27 June 2022)), Picard (https://github.com/broadinstitute/picard (accessed on 28 June 2022)) and GATK (https://github.com/broadinstitute/gatk/releases (accessed on 30 June 2022)) software, with *M. unguiculatus* genome from NCBI (https://www.ncbi.nlm.nih.gov/genome/?term=Meriones+unguiculatus (accessed on 27 June 2022)) as the reference. The TRPV1 gene sequence of *M. meridianus* was assembled by replacing the variant site with SNPs, referring to the genome of *M. unguiculatus*.

### 4.6. Validation of Missense Mutation Sites and Prediction of Protein Structure 

PCR-amplification was performed to verify the mutation sites, and 7 individuals for each species were used to eliminate the intraspecific differences. The PCR reaction volume is totally 25 μL, including 12.5 μL 2 × Taq Master Mix (R312-01, Vazyme), 1 μL forward primer (10 μM), 1 μL reverse primer (10 μM), 2 μL of template cDNA (20 ng), and 8.5 μL ddH_2_O. The PCR reaction was performed on Thermal Cycler (Veriti^TM^ 96-Well 9902, Singapore) while following procedures: Firstly, 95 °C for 3 min, then 95 °C for 15 s, 58 °C for 15 s, and 72 °C for 50 s. These three steps were repeated 35 times, and finally, extension at 72 °C for 5 min. The cDNA was reverse-transcribed from liver total mRNA (5 μg) using cDNA synthesis kit (R312-01, Vazyme). Protein structures of these two analogs were predicted using Swiss-model (https://swissmodel.expasy.org/interactive (accessed on 20 August 2022)). Primers used here were shown in Table S3.

### 4.7. TRPV1 Cloning and Ectopic Expression in E. coli System

According to the nucleotide sequences of TRPV1 in *M. unguiculatus* and *M. meridianus*, specific primers were designed using primer-blast from NCBI. Three pairs of primers were designed to amplify three fragments of TRPV1 in *M. unguiculatus* separately, which have at least 50 base pair (bp) overlapped with each other, covering the whole sequence (Table S4). Then, a full-length sequence was cloned from these three fragments. For TRPV1 in *M. meridianus*, two pairs of primers were designed to obtain the two fragments separately, which were used as template cDNA to amplify the full sequence. Reaction volume is totally 50 μL, 2 × Taq Master Mix (R312-01, Vazyme, Nanjing, China) for 25 μL, 1.5 μL forward primer (10 μM), 1.5 μL reverse primer (10 μM), 3 μL of template cDNA (20 ng), and 19 μL ddH_2_O. Then, the qPCR reaction was performed on Thermal Cycler with the following procedures: 95 °C for 3 min, then 95 °C for 15 s, 58 °C for 15 s, and 72 °C for 50 s, repeating these three steps 35 times, and finally, extension at 72 °C for 10 min. The cDNA reverse-transcription was described in the qPCR experiment.

Full-length coded cDNA sequence was obtained as described above and cloned into pGEM-T easy vector (Promega, Madison, WI, USA). The product was transformed into Trans1-T1 phage-resistant chemically competent cell (CD501, Transgene, Beijing, China), and then the bacteria suspension was coated over the plates and allowed to grow up in 4 °C overnight. Finally, the monoclonal colonies were picked and reproduced in fluid nutrient medium under 25 °C for 7 h. The monoclonal bacterial solution was subsequently sequenced [54].

### 4.8. Statistical Analysis

The data of morphological traits, rectal temperature, and the mRNA expression of TRPV1 gene were analyzed with independent samples *t*-test, and temperature preference were analyzed using two-way ANOVA and independent samples *t*-test, all on SPSS 20.0 (IBM Inc., Chicago, IL, USA). The results are presented as means ± standard error of mean (SEM), and *p* < 0.05 was recognized as a statistical significance. All the figures were processed with Prism 8.0.1 (GraphPad, San Diego, CA, USA).

## 5. Conclusions

In this study, heat sensitivity showed a significant difference between *M. unguiculatus* and *M. meridianus*, but the expression pattern of TRPV1 mRNA represented no discrepancy; however, we identified the specific genetic differentiation of TRPV1 between these two species. Together, our findings supported the hypothesis about the association between genetic diversity in the TRPV1 gene and heat sensitivity phenotypes in *M. unguiculatus* and *M. meridianus*. 

## Figures and Tables

**Table 1:** Morphological traits of *M. unguiculatus* and *M. meridianus*.

Parameters	Species		Statistical Summary
	*M. unguiculatus*	*M. meridianus*	*p*-Value
Body mass (g)	38.7 ± 1.5	48.2 ± 2.7	<0.01
Body length (mm)	101.7 ± 1.8	107.4 ± 2.5	ns
Ear length (mm)	11.9 ± 0.54	15.7 ± 1.27	<0.01
Tail length (mm)	93.8 ± 2.68	100.1 ± 4.33	ns
Metapodial length (mm)	22.6 ± 0.34	26.1 ± 0.83	<0.05

**Table 2:** Summary statistics for TRPV1 diversity in two gerbil species.

Species	Total Length (bp)	Total Variants	Nonsense Variants	Homology of cDNA	Protein Length	Homology of Protein
*M. unguiculatus*	2523	18	2	99.28%	840	99.76%
*M. meridianus*						

**Table 3:** Amino acid mutation sites on TRPV1 in two gerbil species.

	Mutation 1	Mutation 2
*M. unguiculatus*	_100_DSVSSSVEKPPRLYDRRSIF …	… _597_VTLIEDGKNSSVPVEATQHR
*M. meridianus*	_100_DSVSSSVEKLPRLYDRRSIF …	… _597_VTLIEDGKNSSVPMEATQHR

## Data Availability

All data in this study are available upon request from the corresponding authors.

## Supplementary Materials

The following supporting information can be downloaded at: https://www.mdpi.com/article/10.3390/ijms24119123/s1.
