# Supplementary material for: Genetic Diversity of a Heat Activated Channel—TRPV1 in Two Desert Gerbil Species with Different Heat Sensitivity
Source: Int J Mol Sci. 2023 May 23;24(11):9123. doi: 10.3390/ijms24119123 (PMC10252964; doi:10.3390/ijms24119123)
Supplement: Supplementary file 1 [file ijms-24-09123-s001.zip › ijms-2333110-supplementary.docx]

**Supplementary Table S1. Exon sequence of *trpv1* in *M. meridianus*.**

| ATGAAGAAATGGGATAGCTCAGAGTTGGGGGGATCTGACCACCCACCCCAAGAGAACCCCTGCCTGGATCCTCCAGACAGAGACCCTAACTCCAAGCAACCTACAGCCAAACCCCACATTTTCCCCAGCAAAAGCCGTACCCGGCTTTTTGGGAAGGGTGACTCAGAGGAGGCCTCCCCGATGGATTGCCCTTATGAAGAAGGCGGGCTGGCCTCCTGCCCTATAATCACTGTCAGCTCTGTCGTCACCATCCAGAGGCCTGGGGATGGACCTGCCTGTGCCAGGCAGTTGTCCCAGGACTCTGTCTCCAGCAGTGTTGAGAAGCCCCCCAGGCTCTATGATCGCAGGAGCATCTTCGATGCTGTGGCTCAGAGTAACTGCCAGGAGCTAGAGAGCCTGCTGCCCTTCCTGCAGAAGAGCAAGAAGCGCCTGACTGACAGCGAGTTCAAAGACCCAGAGACAGGAAAGACCTGTCTGCTAAAAGCCATGCTCAATCTGCACAATGGACAGAATGACACCATCGCTTTGCTCCTGGACATCGCCCGGCAGACCGACAGCCTGAAGCAGTTCGTCAATGCCAGCTACACAGACAGCTACTACAAGGGTCAGACAGCACTGCACATTGCCATCGAAAGGCGGAACATGGCACTGGTGACCCTCCTGGTGGAGAACGGCGCAGATGTCCAGGCTGCGGCTAATGGCGACTTTTTCAAGAAAACCAAAGGGAGGCCTGGCTTCTACTTTGGTGAGCTGCCTTTGTCCCTGGCTGCATGCACCAACCAGCTGGCCATTGTGAAGTTCCTGCTGCAGAACTCATGGCAGCCGGCGGACATCAGTGCCCGGGATTCGGTGGGCAACACTGTGCTGCACGCCCTGGTGGAGGTGGCAGATAACACGGCTGACAACACCAAGTTCGTGACAAACATGTACAACGAGATCCTGATCCTGGGGGCCAAACTCCACCCCACGCTGAAGCTGGAAGAACTCACCAACAAGAAGGGGCTCACCCCGCTGGCTCTGGCTGCTAGCACTGGGAAGATTGGGGTCTTGGCCTACATTCTCCAGAGGGAGATCCAGGAGCCCGAGTGCCGGCACCTGTCCAGGAAGTTCACGGAATGGGCATATGGGCCTGTGCACTCCTCTCTTTACGACCTGTCCTGCATTGACACCTGCGAGAAGAACTCGGTTCTGGAGGTGATCGCCTACAGCAGCAGCGAGACCCCTAACCGCCATGACATGCTTCTGGTGGAACCTTTGAACCGACTCCTACAGGACAAGTGGGACAGATTTGTCAAGCGCATCTTCTACTTCAACTTCTTCGTCTACTGCTTTTACATGATCATCTTCACCACAGCTGCCTACTATCGGCCTGTGGAAGGCTTGCCCCCCTATAAGCTGAGGAACACCGTTGGAGACTATTTCCGAGTCACTGGAGAGATCCTGTCTGTGTCAGGAGGAGTCTACTTCTTCTTCAGAGGGATTCAGTATTTCCTGCAGAGGCGGCCATCCCTCAAGAGTTTGTTTGTGGACAGCTACAGCGAGATTCTTTTCTTTGTGCAGTCGCTGTTCATGCTGGTGTCTGTGGTACTGTACTTCAGCCAACGCAAGGAGTATGTGGCTTCCATGGTGTTCTCTCTGGCCATGGGCTGGACCAACATGCTCTACTATACCCGAGGGTTCCAGCAGATGGGTATCTACGCTGTCATGATTGAGAAGATGATCCTCCGAGACCTGTGCCGATTTATGTTCGTCTACCTCGTGTTCTTGTTTGGATTTTCCACAGCTGTGGTGACCCTGATCGAGGATGGGAAGAACAGCTCTGTGCCCGTGGAGGCCACGCAGCACAGGTGGCGGGGGCCCGCCTGCAGGTCGCCCGACAACTCCTACAACAGCCTGTACTCCACGTGTCTGGAGCTGTTCAAGTTCACCATCGGCATGGGTGACCTGGAGTTCACTGAGAACTACGACTTCAAGGCCGTCTTCATCATCCTGCTGCTGGCCTACGTGATTCTCACCTACATCCTCCTGCTCAACATGCTCATCGCGCTCATGGGCGAGACCGTCAACAAGATCGCTCAGGAGAGCAAGAACATCTGGAAGTTGCAGAGAGCCATCACCATCCTGGATACAGAGAAGAGCTTCCTGAAGTGCATGAGGAAGGCCTTCCGCTCTGGCAAGCTGCTGCAGGTGGGCTTCACACCTGATGGCAAAGATGACTACCGGTGGTGTTTCAGGGTGGACGAGGTCAACTGGACTACGTGGAACACCAACGTGGGCATCATCAATGAGGACCCAGGCAACTGTGAGGGCGTCAAGCGTACCCTGAGCTTCTCCCTGAGGTCAGGCCGAGTTTCAGGGAGAAACTGGAAGAACTTTGCCCTGGTTCCCCTTCTGAGGGATGCAAGCACTCGAGATAGGCATACCACCCAGCCCGAGGAAGTTCATCTGAAGCATTTTGCAGGGTCCCTTAAGCCAGAGGATGCTGAGGTCTTCAAGGATTCCATGGCCCCAGGAGAGAAGTGA |
| --- |

**Supplementary Table S2. The gene specific primers for qPCR.**

| Gene | Forward Primer (5’-3’) | Reverse Primer (5’-3’) |
| --- | --- | --- |
| TRPV1  GAPDH | GATGGGCATCTACGCTGTCA  AGGTCGGTGTGAACGGATTTG | ATCCTCGATCAGGGTCACCA  TGTAGACCATGTAGTTGAGGTCA |

**Supplementary Table S3. The primers for mutation validation**

| Gene | Forward Primer (5’-3’) | Reverse Primer (5’-3’) |
| --- | --- | --- |
| Site 1  Site 2 | GGTCAGGGCTAGGGTAGAGA  TTGGTTTCCATGCACTCCACC | CTGTCAGTCAGGCGCTTCTT  GTTGTAGGAGTTGTCGGGCG |

**Supplementary Table S4. The gene specific primers for *trpv1* cloning.**

| Gene | Forward Primer (5’-3’) | Reverse Primer (5’-3’) |
| --- | --- | --- |
| *utrpv1* Frag1 | ATGAAGAAATGGGATAGCTCAGAGTTGG | CCGATAGTAGGCAGCTGTGG |
| *utrpv1* Frag2 | CATATGGGCCTGTGCACTCC | TCACTTCTCTCCTGGGGCCATG |
| *mtrpv1* Frag1 | ATGAAGAAATGGGATAGCTCAGAGTTGG | CCCCAGGATCAGGATCTCGT |
| *mtrpv*1 Frag2 | GACATCAGTGCCCGGGATTC | GCACAGAGCTGTTCTTCCCA |
| *mtrpv*1 Frag3 | GCTGTGGTGACCCTGATCGAGG | TCACTTCTCTCCTGGGGCCATG |

*utrpv1*, *trpv1* of *M. unguiculatus*; *mtrpv*1, *trpv1* of *M. meridianus*
